# Enhanced solar light photocatalytic degradation of tetracycline by aero-GaN and ZnO microtetrapods functionalized with noble metal nanodots

**DOI:** 10.1016/j.heliyon.2024.e40989

**Published:** 2024-12-06

**Authors:** Vladimir Ciobanu, Tatiana Galatonova, Pavel Urbanek, Tudor Braniste, Florica Doroftei, Milan Masar, Pavol Suly, Veaceslav Ursaki, Barbora Hanulikova, Tomas Sopik, Vladimir Sedlarik, Ivo Kuritka, Ion Tiginyanu

**Affiliations:** a Centre of Advanced Research in Bionanoconjugates and Biopolymers, “Petru Poni” Institute of Macromolecular Chemistry, 41A, Grigore Ghica Voda Alley, 700487, Iasi, Romania; b National Centre for Materials Study and Testing, Technical University of Moldova, 168, Stefan cel Mare av., 2004, Chisinau, Republic of Moldova; c Centre of Polymer Systems, Tomas Bata University in Zlin, 5678, tr. Tomase Bati, CZ 76001, Zlin, Czech Republic; d Academy of Sciences of Moldova, 1, Stefan cel Mare av., 2001, Chisinau, Republic of Moldova

**Keywords:** Photocatalysis, Tetracycline, GaN, ZnO, Metal nanodots

## Abstract

The escalating global problem of antibiotic contamination in wastewater demands innovative and sustainable remediation technologies. This paper presents a highly efficient photocatalytic material for water purification: a three-dimensional ultra-porous structure of interconnected GaN hollow microtetrapods (aero-GaN), its performance being further enhanced by noble metal nanodot functionalization. This novel aero-nanomaterial achieves more than 90 % of tetracycline degradation within 120 min under UV and solar irradiation, demonstrating its effectiveness in both static and dynamic flow conditions, with the potential for reuse and recyclability. The higher surface area and chemical stability of the 3D aero-GaN architecture, compared to analogous ZnO structures, establish its significant potential for advanced water treatment applications and filter technologies.

## Highlights

• Ultra-porous aero-GaN show efficient photocatalytic performances similar to those inherent to ZnO microstructures.
• Aero-GaN micro-nanoarchitectures are stable, reusable, and degrade tetracycline under UV and solar light irradiation.
• Ag or Pt functionalized aero-GaN and ZnO microtetrapods boost the photocatalysis reducing tetracycline degradation time.

## Graphical abstract

**Figure:**
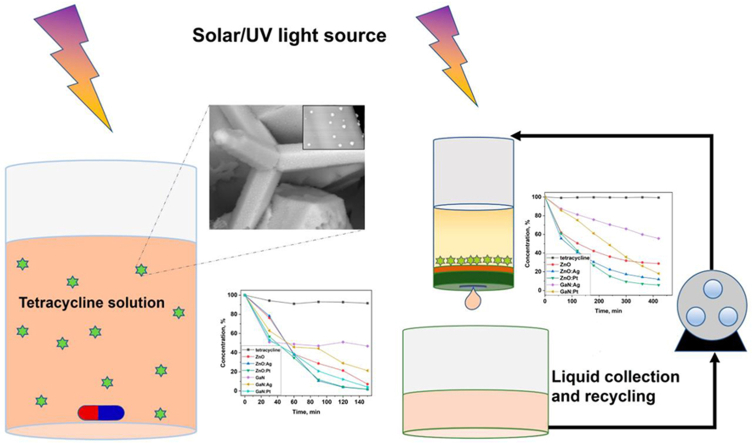

## 1 Introduction

Water pollution from various sources, particularly from the pharmaceuticals used for the treatment of diseases, presents a critical environmental challenge due to the discharge of pharmaceutical compounds in aquatic systems. The excessive use of antibiotics and their derivatives in medicine and agriculture is a significant concern as it contributes to the rise of antibiotic-resistant bacteria and poses risks to aquatic life and human health. The discharge of pharmaceutical compounds, including antibiotics like tetracycline, originates from multiple sources such as manufacturing processes, hospitals and home-cured patients, and improper disposal of unused medications [1]. Tetracycline represents a broad-spectrum antibiotic which is extensively utilized in both human and veterinary medicine, contributing substantially to the pollution of the aquatic environment. Its adverse effects on ecosystems and human health necessitate effective strategies for its removal [2].

The pursuit of new photocatalytic materials is driven by the need to overcome limitations of existing materials, expand the range of applications, and enhance both efficiency and sustainability. In recent years, semiconductor nanomaterials like Zinc Oxide (ZnO) and Gallium Nitride (GaN) have drawn attention due to their photocatalytic properties, exhibiting promising results under both visible and ultraviolet (UV) light irradiation due to their suitable bandgap structures, efficient charge carrier separation and the possibility to fabricate high surface area structures. The use of ZnO and GaN as photocatalysts for degrading various pollutants, including antibiotics, has shown considerable potential. Their ability to generate reactive oxygen species (ROS) upon light irradiation enables the degradation of organic pollutants through oxidation processes [3,4]. GaN shows high chemical stability in various environmental conditions [5], while ZnO stability in liquids is strongly influenced by the solution pH [6]. Zinc oxide is a relatively cheap material, easy to fabricate in a variety of shapes and possesses very good optical properties, nevertheless the material usage in practical applications is limited due to its poor chemical stability. The enhanced stability of GaN renders it more resilient to chemical degradation, making the material a promising candidate for prolonged photocatalytic applications in water treatment scenarios. Modification of the semiconductor surface with other species, such as metal nanoparticles, carbon structures or other metal oxide semiconductor nanoparticles, can increase the stability and catalytic activity of the pristine materials [7,8].

The increase of the active surface area represents one of the most important strategies when developing materials for use as photocatalysts. 3D structures like ZnO microtetrapods proved to be efficient for environmental applications. ZnO microtetrapods showed good photocatalytic activity by degrading dyes such as Methylene Blue [9,10], Rhodamine B [11], or other organic compounds like 2,4-dinitrophenol [12], Bisphenol A [13] and others.

ZnO microtetrapods can serve as sacrificial templates in the process of fabrication of other 3D nanostructures resulting in the formation of a new class of highly porous materials, called aeromaterials [[14], [15], [16]]. Due to the inherent high specific surface area, these materials are promising candidates for photocatalytic applications in e.g. water treatment or photoactivated air filters.

Considering the impact of pharmaceutical pollution on water-living organisms, the potential of semiconductor nanomaterials based on ZnO and GaN in photocatalytic degradation can contribute to the development of sustainable strategies for water treatment. Current photocatalysts often suffer from rapid electron-hole recombination, which reduces their efficiency. Using metal nanodots and, specifically, noble metal nanodots, to functionalize semiconductors enables one to improve photocatalytic properties through enhanced light absorption, charge separation, and suppressed carrier recombination. The combination of a semiconductor with metal nanodots creates a synergistic effect. The semiconductor provides the initial light absorption and charge generation, while the metal nanodots enhance light absorption, improve charge separation, and provide additional catalytic activity, leading to substantially improved photocatalytic efficiency and broader applicability. Thus, our goal is to explore the utilization of the aero-GaN unique nanoarchitecture functionalized with Ag or Pt nanodots as solar light-driven photocatalyst for effectively degrading tetracycline, emphasizing its potential role in mitigating pharmaceutical pollution and advancing water purification technologies. The results are compared to those inherent to ZnO microtetrapods which served as sacrificial templates in the aero-GaN fabrication process.

## 2 Materials and methods

### 2.1 Synthesis of aero-GaN

Aero-GaN was obtained by growing thin layers of gallium nitride using Hydride Vapor Phase Epitaxy (HVPE) on sacrificial templates consisting of ZnO microtetrapods. The ZnO microtetrapods, obtained by Flame Transport [17] were synthesized at Kiel University in Germany and kindly provided by Prof. Rainer Adelung. For the HVPE process metallic gallium (Ga), ammonia (NH_3_), and hydrogen chloride (HCl) were used as precursors, and hydrogen (H_2_) as transport gas. The growth process takes place in a horizontal quartz reactor with multiple zones of temperature. First, in the source zone, metallic gallium interacts with hydrogen chloride at 800 °C, then, in the reaction zone, at 650 °C, the process of GaN deposition occurs for 10 min leading to the formation of the nucleation layer. The growth of the GaN layer takes place in the third step, at 850 °C during 10 min [16]. Simultaneously, at high temperature and in the harsh environment inside the reactor, the ZnO template is decomposed, finally resulting in the formation of hollow GaN microtetrapods with a wall thickness of around 80 nm. The aero-GaN structure contains an ultrathin layer of ZnO that remains on the inner surface of the microtubes even after an additional treatment of sample at high temperatures in highly corrosive environment of H_2_ and HCl [18].

### 2.2 Modification of materials with noble metals

ZnO and hollow GaN microtetrapods were functionalized with Ag or Pt by physical deposition of about 5 nm noble metals from Ag or Pt targets in a Cressington Sputter Coater 108Auto, followed by a thermal treatment at 300 °C for 1 h in air conditions, the formation of nanodots taking place due to surface tension and minimization of surface energy.

### 2.3 Materials characterization

The morphology of the fabricated materials was investigated by Nova NanoSEM 450 (The Netherlands, FEI company) and Verios G4 UC (Thermo Scientific, Brno, Czech Republic) scanning electron microscopes. Images with topographic contrast were taken using an in-column secondary electrons detector (TLD), and the material contrast and elemental analysis were studied with a Circular Backscatter Detector (CBS) at accelerating voltages of 5 kV and 15 kV, respectively. The working distance up to the sample was 4 mm for the TLD detector and 5 mm for CBS. The elemental microanalysis was performed by the Octane SSD (area 30 mm^2^) EDX (energy-dispersive X-ray) detector (AMETEC, Inc).

The X-ray diffraction patterns were collected using a Rigaku Miniflex-600 powder diffractometer equipped with a Co Kα source radiation (λ = 1.7903 Å) operating at 40 kV and an emission current of 15 mA. Diffraction patterns were recorded in the diffraction angle 2θ ranging from 20 to 90° at the scanning speed of 6°/min step 0.2°.

A PerkinElmer 1050 UV/Vis spectrophotometer was used to study the absorption spectra of the solutions, liquid dispersions, and for determination of tetracycline concentration.

The Raman spectra were obtained with the Raman microscope Nicolet DXR (Thermo Scientific, USA). The spectra were collected with the excitation laser beam of 532 nm, 20 expositions and an exposition time of 4–6 s.

Nitrogen adsorption/desorption isotherms were recorded with a volumetric gas adsorption analyzer (BELSORP Mini II, BEL Japan) at 77 K. Prior to taking measurements, the samples were degassed in sample cells at 400 °C for 4 h. The specific surface area (SSA) was determined with multi-point Brunauer–Emmett–Teller (BET) analysis, applying at least five data points within a relative pressure range of 0.05–0.20 *p*/*p*_0_ (Data Analysis Software, version 6.4.1.0, MicrotracBEL Corp, Japan).

HPLC analysis was performed on a 1260 Infinity LC system (Agilent Technologies, Santa Clara, USA). Chromatographic separation of the components of the samples was carried out on a ZORBAX Extend C18 column (50 mm × 2.1 mm, 1.8 μm) (Agilent Technologies, Santa Clara, USA) at a flow rate of 0.300 mL/min that was maintained at 30 °C.

Detection was performed on a quadrupole time of flight mass spectrometer (6530 Q-TOF, Agilent Technologies, Santa Clara, USA) employing an electrospray ion (ESI) source set to positive mode. Mass spectra were acquired over the *m*/*z* 100–1500 range at a scan rate of 3 scan·s^−1^. Accurate mass measurements were obtained via a calibration solution involving the use of internal reference masses (purine (C_5_H_4_N_4_) at *m*/*z* 121.050873, and HP-0921 [hexakis-(1H,1H,3H-tetrafluoropentoxy)-phosphazene] (C_18_H_18_O_6_N_3_P_3_F_24_) at *m*/*z* 922.009798).

### 2.4 Photocatalytic activity of ZnO and aero-GaN microtetrapods

The tetracycline used in the experiments was acquired from Sigma Aldrich (CAS number 60548). The photocatalytic investigations were performed under both UV and visible light irradiation in two different configurations. In the first case, 5 mg of material was mixed with 10 ml of tetracycline solution with a concentration of 10 mg/L. The solution was continuously mixed with a stirrer at 250 rpm while being irradiated laterally with visible light from a Solar Simulator Pico Solar G2V using the standard Solar Spectrum AM1.5g with a power density of 87.5 mW/cm^2^, or irradiated from the top with a Focused UV lamp type C-10A-HE with power density of 3.4 mW/cm^2^. In both cases the solution temperature was increased not more than 2 °C. A 1 ml solution was extracted every 30 min, centrifuged twice at 6k rcf for 5 min to avoid the presence of the material in the suspension and then the absorbance spectra were collected. In the second configuration, the tetracycline degradation was investigated under liquid flow conditions, by using a peristaltic pump with the set flow condition of 2.5 ml/min and irradiated with the power density of 3.2 mW/cm^2^ by the UV lamp. The concentration of tetracycline was determined from the absorption spectra at 270 nm. First, the calibration curve was performed, then the concentration was determined from the linear regression curve. The absorption spectra were taken in PMMA cuvettes.

## 3 Results and discussion

**Fig. 1:**
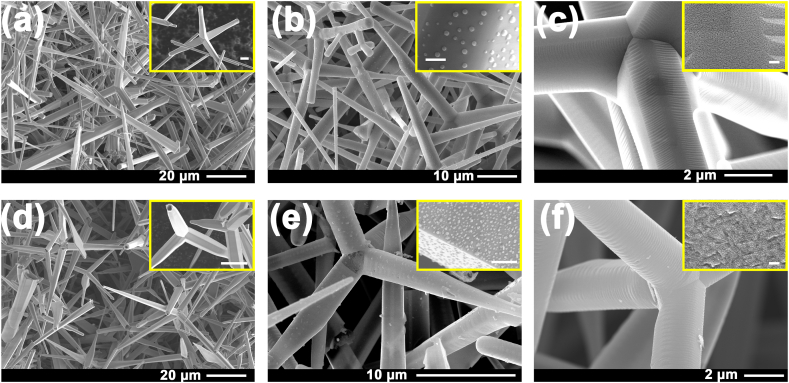
SEM images of ZnO pristine microtetrapods (a), and ZnO functionalized with Ag (b) and Pt (c) nanodots; and of aero-GaN (d) and aero-GaN functionalized with Ag (e) and Pt (f) nanodots. (The scalebar of the insert in a and d is 5 μm; in b and e is 400 nm; and in c and f is 50 nm).

The morphologies of the ZnO and aero-GaN microtetrapods are illustrated in Fig. 1. The dimensions of the ZnO microtetrapods arms vary in the range of 5–40 μm in length and 1–5 μm in diameter. The resulting aero-GaN hollow microtetrapods have a wall thickness of the tubes of about 80 nm. According to EDX measurements, some traces of ZnO (around 1.5 at.%) can still be visible after the sacrificial template is removed, which can be attributed to the presence of a chemically stable film of ZnO on the inner surface of the tubes, or due to the formation of the solid solution phase (Ga_1-x_Zn_x_)(N_1-x_O_x_) [18].

Functionalization of the ZnO with 7 nm of Ag or Pt layers, followed by the annealing process at 300 °C, results in the formation of Ag nanodots with diameter from 90 nm to 350 nm, or Pt nanodots with diameter from 3 nm to 20 nm, respectively. The EDX analysis confirms the presence of around 0.2 at.% Ag and about 2 at.% Pt on the selected areas.

Functionalization of aero-GaN with Ag or Pt results in the formation of nanodots with the size in the range from 3 to 25 nm in case of Pt and clusters of nanodots with dimensions of 25–150 nm in case of Ag. The EDX analysis of functionalized GaN-aeromaterial confirms the presence of about 0.25 at.% Ag.

**Fig. 2:**
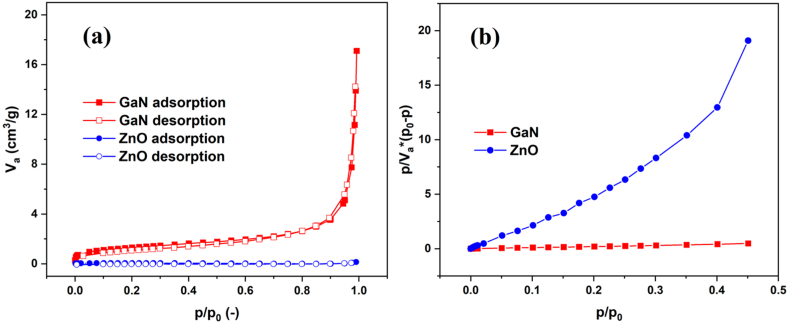
Nitrogen adsorption–desorption isotherms (a) and corresponding BET plots (b) of the investigated photocatalysts (color should be used for figure in print).

The specific surface area (SSA) of the photocatalysts was estimated by nitrogen adsorption–desorption (Fig. 2a) according to BET (Brunauer, Emmett and Teller) analysis method (Fig. 2b). As can be seen, both ZnO and aero-GaN investigated photocatalyst materials correspond to adsorption isotherm type II according to IUPAC (International Union of Pure and Applied Chemistry) classification [19]. Based on the observed results, the investigated materials can be considered as nonporous materials. ZnO microtetrapods possess a specific surface area of 0.2 m^2^/g, while in case of aero-GaN, it increased up to 4.7 m^2^/g, which also corroborates with the mass change of the material before and after epitaxial growth of GaN.

More results from nitrogen adsorption-desorption analysis are summarized in Table 1. The summarized data include values such as total specific area (A_S, BET_), monolayer volume (V_m_), and energy constant of the first layer (C). As already mentioned, SSA was determined with a multi-point BET analysis method using at least five data points within a relative pressure (p/p_0_) range from 0.05 to 0.20. More information can be obtained according to the energy constant. The optimum value for the mesopores is from 0 to 200. The values of C < 0 or C > 200 indicate the presence of micropores. Significant differences were also obtained in other characteristics such as monolayer volume and total pore volume at normal pressure (p/p_0_ ∼ 1), where these values of GaN are significantly higher (by two orders of magnitude) in comparison to values of ZnO microtetrapods. The smaller sizes of pores can be also visible from SEM images shown in Fig. 1.

**Table 1:** Evaluation of specific surface area of investigated photocatalysts.

Sample	ZnO	Aero-GaN
A_S,BET_ [m^2^/g]	0.2	4.7
V_m_ [cm^3^ (STP)/g ]	4.64·10^−2^	1.08
Total pore volume at p/p0 = 0.990 [cm^3^/g]	2.11·10^−4^	2.28·10^−2^
Mean pore diameter [nm]	4.2	19.4

**Fig. 3:**
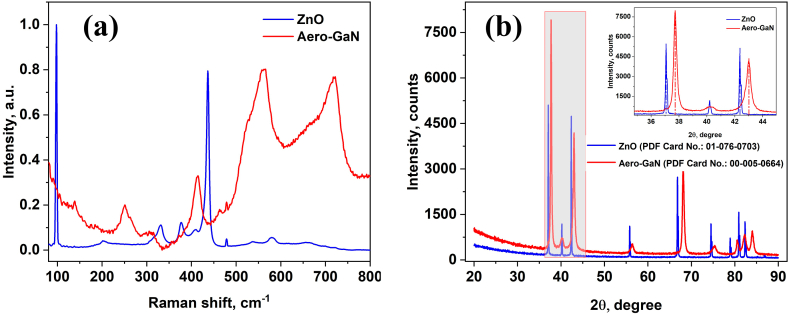
Raman (a) and XRD (b) spectra of ZnO and aero-GaN microtetrapods (color should be used for figure in print).

The Raman spectra of the investigated materials are shown in Fig. 3a. The well-defined peaks can be observed for the hexagonal wurtzite structure of ZnO. The Raman scattering peaks at 98 cm^−1^, 377 cm^−1^, 408 cm^−1^, 437 cm^−1^ and 583 cm^−1^ are attributed to the first order E_2_^low^, A_1_(TO), E_1_(TO), E_2_^high^ and A_1_(LO)/E_1_(LO) Raman active modes of the wurtzite ZnO structure, respectively. The peaks at 202 cm^−1^ and 331 cm^−1^ have been attributed to the second order Raman process involving 2E_2_^low^ and E_2_^high^ - E_2_^low^ modes, respectively [20].

The Raman spectrum from the aero-GaN [21] material is dominated by the E_2_^high^ and A_1_(TO) peaks from the wurtzite GaN at 564 and 729 cm^−1^, respectively, with some peaks from the ZnO traces.

The XRD pattern of the ZnO template (Fig. 3b) demonstrates the high crystalline quality of the material, all the peaks being indexed to the hexagonal wurtzite phase in good agreement with the data from JCPDS card No. 00-005-0664 [22]. No other peaks have been detected, implying that the ZnO template contains no spurious phases. The same is true for the aero-GaN material, only peaks related to the hexagonal wurtzite phase of GaN being observed in the XRD pattern, according to the data from JCPDS card No. 01-076-0703. The peaks in the GaN pattern are shifted to higher angle values as compared to the ZnO pattern, and the full width at half maximum (FWHM) is larger. The larger width of peaks suggests that the aero-GaN material is composed of smaller size GaN nanograins.

**Photocatalytic performance**

**Fig. 4:**
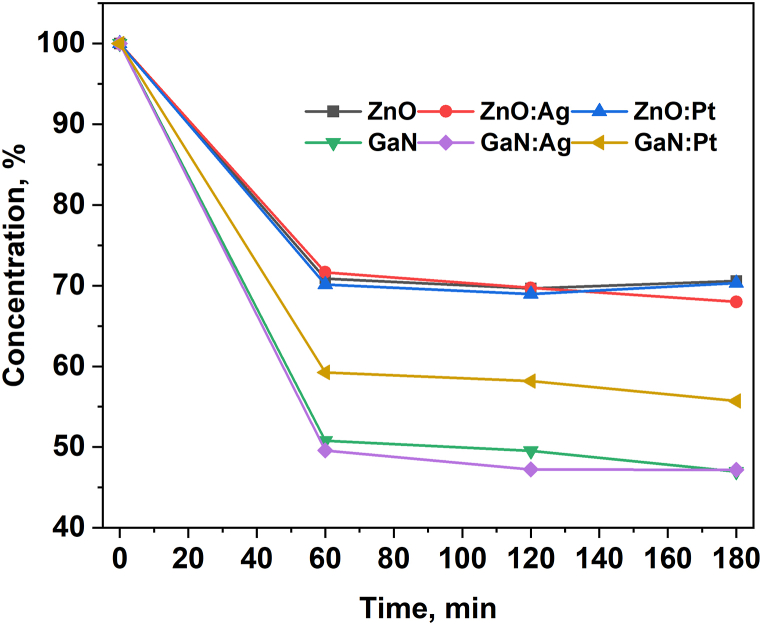
Adsorption of the tetracycline on the surface of materials under dark conditions (color should be used for figure in print).

Tetracycline degradation was assessed by measuring the optical absorption at 270 nm. The photocatalytic activity of the materials was evaluated by the degradation of tetracycline solution under irradiation with visible or UV light at room temperature. As light sources, a Solar Simulator (standard spectrum AM1.5g) and a focused UV lamp (λ = 365 nm) were used. The visible light intensity used in the experiments equals 87.5 mW/cm^2^ in the case of visible light and 3.4 mW/cm^2^ in the case of UV irradiation. The blank test on the sample denoted as TCRS (tetracycline reference sample) confirmed that the photolysis was negligible without the catalyst, indicating the absence of self-sensitized photodegradation of the antibiotic. The tested samples are denoted according to the material code of the dispersed photocatalyst (ZnO stands for ZnO microtetrapods; ZnO:Ag and ZnO:Pt stand for ZnO microtetrapods decorated by silver and platinum nanodots, respectively; GaN stands for aero-GaN microtetrapods; GaN:Ag and GaN:Pt stand for aero-GaN microtetrapods decorated by silver and platinum nanodots, respectively). The adsorption of tetracycline on the material surface was monitored under dark conditions for the same time interval as for the other experiments. It was observed that materials have different adsorption rates and saturated capacity, as can be seen from Fig. 4.

ZnO microtetrapods, with or without functionalization with metal nanodots, absorb around 30 % of tetracycline from the solution under test during the first hour and then this concentration remains constant. GaN-aeromaterial, on the other hand, has a higher adsorption rate, that may be related to its higher surface area. The as-grown aero-GaN as well as samples functionalized with Ag show an adsorption of around 53 % of tetracycline, while the aero-GaN functionalized with Pt exhibits adsorption of about 45 % of tetracycline during the first hour and then this concentration remains constant.

The kinetics of the tetracycline photodegradation was fitted according to the pseudo-first-order model:

$$\ln\left(\frac{C_{0}}{C_{t}}\right)=kt\text{,}$$

where, *C*_0_ and *C*_*t*_ represent the concentrations of tetracycline in solutions at irradiation time *t* = 0 min and *t*, respectively.

**Fig. 5:**
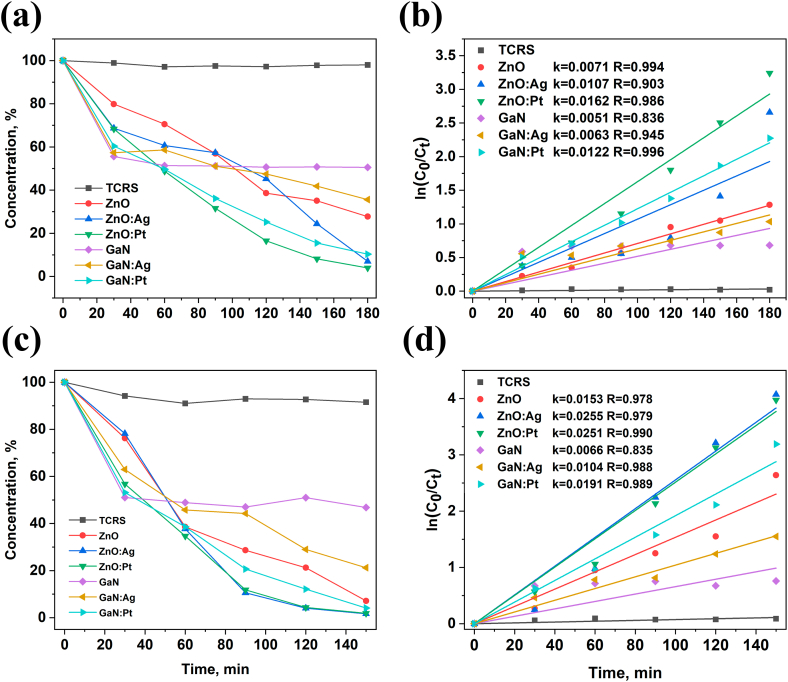
Photocatalytic degradation of tetracycline by ZnO and GaN hollow microtetrapods under solar light (a) and UV (c) irradiation and their pseudo-first-order kinetic model (b, d) (color should be used for figure in print).

Upon irradiation with the Solar Simulator, the tetracycline in TCRS is not influenced at all as well as in the presence of aero-GaN in the solution, only the adsorption on the material surface being observed. When aero-GaN is functionalized, the tetracycline is degraded up to 65 % and 90 % in case of Ag or Pt nanodots functionalization, respectively, the degradation rate being 0.012 min^−1^ for the latter material, as shown in Fig. 5b.

Pure ZnO microtetrapods proved to be efficient by degrading around 72 % of tetracycline, while ZnO functionalized with Ag and Pt nanodots showed a decomposition of tetracycline during 180 min of about 93 % and 96 %, respectively. Functionalization of ZnO with Pt nanodots leads to an increase of the degradation rate up to 0.016 min^−1^.

In case of irradiation by UV light (Fig. 5c), the tetracycline concentration in the control group in TCRS decreases slightly by about 7 %. Aero-GaN showed a decrease of tetracycline of about 47 %, which is similar to the concentration decrease in case of visible light irradiation or in dark conditions, which demonstrates not only the adsorption of the tetracycline on its surface, but also a strong adhesion force which could be the reason for protection of the tetracycline upon UV light irradiation. On the other hand, aero-GaN functionalized with Ag or Pt nanodots became efficient by degrading 80 % and 95 % of tetracycline within 2 h, with a maximum degradation rate of 0.019 min^−1^, as illustrated in Fig. 5d.

ZnO and modified ZnO microtetrapods proved to be efficient by degrading more than 90 % of tetracycline during 120 min (0.015 min^−1^ < k < 0.025 min^−1^). The obtained results show better performance as compared to other materials from literature such as CuInS_2_ quantum-dot-modified g-C3N4 S-scheme heterojunction photocatalyst [23], or similar performance as WO_3_–ZnO/AC composite material that shows similar time for degrading more than 90 % of tetracycline [24].

The material decoration with noble metal nanodots resulted in the reduction of the optical transmission of both ZnO and GaN aeromaterials, throughout the visible region, probably due to the Localized Surface Plasmon Resonance (LSPR), which is advantageous for enhancing the photocatalytic activity. It is known that doping or functionalization with metals could also mean a decrease in the optical bandgap, suggesting an enhanced ability to capture the visible light, which facilitates a large amount of charge carrier generation, thereby improving the photocatalytic ability. The measured PL spectra, not illustrated in the article, show reduced signals from the functionalized materials as compared to those inherent to the pristine ZnO or GaN, as well as an increase of the average decay time determined from the time resolved photoluminescence measured at the excitation energy of 1.95 eV and 1.82 eV, respectively, indicating a higher recombination time [25,26]. The lower PL intensity exhibited by GaN and ZnO micro-nanoarchitectures functionalized by metal nanodots is consistent with their higher photocatalytic activity. This is attributed to the suppressed charge carrier recombination in the functionalized materials due to the presence of Ag and Pt nanoparticles which act as electron traps, facilitating efficient charge separation and reducing free carrier recombination. Thus, the enhanced photocatalytic activity of functionalized GaN and ZnO architectures is explained by the efficient charge separation resulting from the interaction between noble metal nanoparticles and semiconductor (GaN or ZnO) microstructures, reducing charge carrier recombination [23,[27], [28], [29], [30], [31]].

To demonstrate the possibility to recycle and continuously use of the materials, a special setup was elaborated, where the photocatalysis tests were performed under solution flow condition and under UV irradiation with an UV lamp with the power density of 3.1 mW/cm^2^. The flow speed was established at 2 ml/min using a peristaltic pump. A Whatman filter with a retention of 8–10 μm particles was used, and the solution transparency was investigated every 60 min for 7 h, which is considered as one complete cycle. For these photocatalytic tests, pristine aero-GaN was excluded, as it demonstrated only the adsorption of antibiotics on its surface and protection against degradation.

**Fig. 6:**
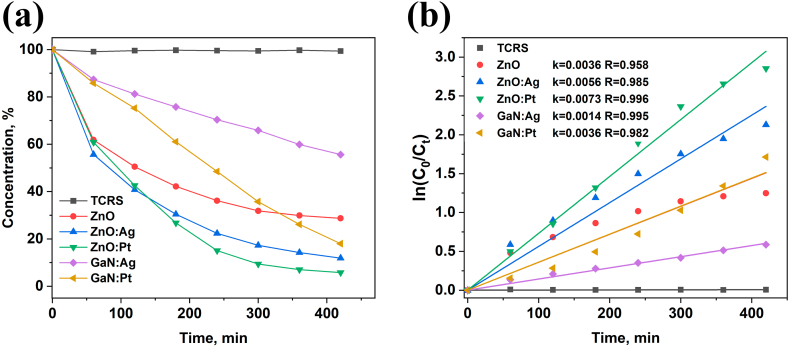
Photocatalytic degradation of tetracycline by ZnO and GaN hollow microtetrapods under liquid flow condition and UV irradiation (a) and the applied kinetic model (b) (color should be used for figure in print).

The photocatalytic tests were performed in 3 cycles. The concentration of the tetracycline in the control sample without any catalyst (also denoted TCRS in the figures) was constant, a decrease of only around 2 % being observed. Functionalization of aero-GaN with Ag dots leads to a decrease of about 45 % of tetracycline, whereas modification with Pt degraded about 72 % of the antibiotic, with the degradation rate of 0.001 min^−1^ and 0.003 min^−1^, respectively (Fig. 6).

ZnO microtetrapods and their functionalization, on the other hand, proved to be efficient by degrading 71 % of tetracycline by pristine ZnO, 88 % in case of Ag functionalized ZnO microtetrapods and 95 % in case of using Pt (0.003 min^−1^ < k < 0.007 min^−1^).

A slight decrease of activity during 3 cycles experiment was observed in the case of aero-GaN:Ag sample, where the efficiency decreased from 45 % to 38 %, whereas the sample consisting of ZnO microtetrapods functionalized with Ag nanodots proved to be even more efficient by completely degrading tetracycline during 7 h, the rest of materials were efficient as in the case of first cycle.

**Fig. 7:**
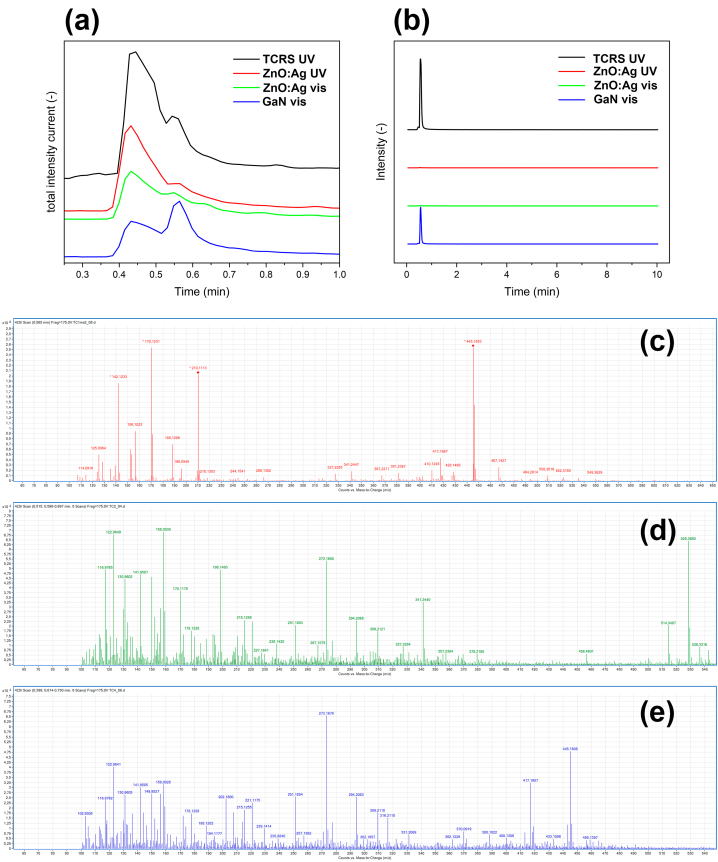
HPLC-MS analysis of the samples after photodegradation: (a) TIC (total ion current) chromatograms and (b) corresponding chromatograms for the *m*/*z* = 445.1583 channel, characteristic for the tetracycline molecular ion [M+H]^+^. HPLC-MS spectra of the tetracycline control sample TCRS (c), and of tetracycline after photocatalysis with the ZnO:Ag UV (d) and GaN (e) under visible light irradiation (color should be used for figure in print).

The degradation of tetracycline was also verified by HPLC-MS analysis shown in Fig. 7. The graph in Fig. 7a shows total ion current chromatograms of the TCRS and three selected samples after irradiation. The sampling was performed at moments corresponding to the ending points of photodegradation dependencies in Fig. 5 (i.e. TCRS, aero-GaN and ZnO:Ag solar in Fig. 5a, while ZnO:Ag UV in Fig. 5c). The first large peak at the elution time 0.45 min in all chromatograms corresponds to poorly separated low molecular species present in the tested solutions. Nevertheless, the second peak at the elution time 0.56 is well separated and is related to the tetracycline molecule. The chromatograms in Fig. 7b clearly indicate unambiguously the presence of tetracycline and at this elution time showing the chromatogram of the total ion current at *m*/*z* = 445.1583 channel, corresponding to the isotopic peak of tetracycline [M+H]^+^. The data for TCRS shows no degradation under UV and for pristine aero-GaN is manifested only adsorption on the surface of the material manifested by the decrease of signal intensity at *m*/*z* = 445.1583 in comparison to TCRS. On the other hand, full degradation of tetracycline was proven when using functionalized ZnO microtetrapods with Ag nanodots not only under UV light but also under visible light irradiation.

The studied literature provided us with an outline of possible degradation products, but the different experimental setups do not allow us to obtain identical results as, for example, with those of the authors [[32], [33], [34]]. Exemplary results of MS analysis are given in Fig. 7c-e. According to Ref. [32], the benzene ring of tetracycline (*m*/*z* = 445) is hydroxylated by the hydroxyl radicals to produce an intermediate isomer and product. In this work, we were able to identify the following degradation products: *m*/*z* = 433; 211 and 362. Besides the *m*/*z* peaks of tetracycline, several *m*/*z* peaks at 158; 198; 273; 294; 309; 316; 341; 433; 456; 514; 528 and 536 were observed in the analyzed spectra, suggesting the formation of other intermediates. Elucidation of the structure of these products was not the goal of this work and may be clarified in further follow-up work [[32], [33], [34]].

## 4 Conclusion

In this work, we demonstrated the fabrication of a new photocatalyst material consisting of aero-GaN hollow microtetrapods functionalized by noble metal (silver and platinum) nanodots with photocatalytic activity towards tetracycline model pollutant under UV and solar AM1.5g radiation. The Raman and XRD studies confirmed a good crystallinity phase of the studied materials. BET analyses have shown an increase of the active surface area by about 20 times for the aero-GaN as compared to that inherent to ZnO which was used as sacrificial material for the fabrication of GaN hollow microtetrapods. The larger surface area of aero-GaN proved a better adsorption of the tetracycline on its surface as demonstrated by dark adsorption experiments. The photocatalytic activity aero-GaN and ZnO microtetrapods was observed under both UV and solar radiation being enhanced by silver and even more by platinum nanodot decoration. Whereas ZnO is known to suffer from corrosion, the application of the stable GaN material resulted in efficient photocatalysts as well. The photocatalytic activity of pristine aero-GaN was not significant, being overweight by its adsorption ability. Nevertheless, the photocatalytic performance under both UV and solar light was strongly enhanced by functionalization of aero-GaN with silver and even more by platinum nanodots. Both sets of materials scored well not only as powders dispersed in the liquid volume but also immobilized on a Whatman filter surface in a drip-flow-reactor. The full degradation of tetracycline was proven by the UV/Vis spectroscopy and by HPLC-MS techniques.

## CRediT authorship contribution statement

**Vladimir Ciobanu:** Writing – review & editing, Writing – original draft, Methodology, Investigation, Formal analysis, Conceptualization. **Tatiana Galatonova:** Writing – review & editing, Investigation, Data curation. **Pavel Urbanek:** Writing – review & editing, Visualization, Project administration, Methodology, Investigation. **Tudor Braniste:** Writing – review & editing, Methodology, Conceptualization. **Florica Doroftei:** Writing – review & editing, Investigation. **Milan Masar:** Writing – review & editing, Investigation. **Pavol Suly:** Writing – review & editing, Investigation. **Veaceslav Ursaki:** Writing – review & editing, Validation, Formal analysis. **Barbora Hanulikova:** Writing – review & editing, Investigation. **Tomas Sopik:** Writing – review & editing, Investigation. **Vladimir Sedlarik:** Writing – review & editing, Resources, Project administration, Funding acquisition. **Ivo Kuritka:** Writing – review & editing, Visualization, Validation, Supervision, Project administration, Methodology, Conceptualization. **Ion Tiginyanu:** Writing – review & editing, Supervision, Resources, Project administration, Funding acquisition, Conceptualization.

## Data availability

Data will be made available on request.

## Declaration of competing interest

The authors declare that they have no known competing financial interests or personal relationships that could have appeared to influence the work reported in this paper.
